# Properties of Selected Additive Materials Used to Increase the Lifetime of Tools for Crushing Unwanted Growths Using Hardfacing by Welding Technology

**DOI:** 10.3390/ma18133188

**Published:** 2025-07-05

**Authors:** Miroslava Ťavodová, Monika Vargová, Dana Stančeková, Anna Rudawska, Arkadiusz Gola

**Affiliations:** 1 Faculty of Technology, Technical University in Zvolen, Študentská 26, 960 01 Zvolen, Slovakia; tavodova@tuzvo.sk (M.Ť.); monika.vargova@tuzvo.sk (M.V.); 2 Faculty of Mechanical Engineering, University of Žilina, Univerzitná 1, 010 26 Žilina, Slovakia; dana.stancekova@fstroj.uniza.sk; 3 Faculty of Mechanical Engineering, Lublin University of Technology, Nadbystrzycka 36, 20-618 Lublin, Poland; a.rudawska@pollub.pl

**Keywords:** mulching forestry tools, hardfacing, wear-resistance, microstructure, hardness

## Abstract

This article focuses on the possibilities of increasing the service life of tools for crushing unwanted growths. One way to increase their service life is to increase the hardness and resistance to abrasive wear of exposed surfaces of the tool, which are their face and back. At the same time, however, care must be taken to ensure that the shape and weight of the tool is not altered after the additive has been hardfaced on. Thus, the tool was first modified by removing the material by milling from the face and back. Subsequently, two surfacing materials, namely UTP 690 and OK WearTrode 55, were chosen and hardfaced by welding onto the pre-prepared surfaces. After hardfacing by welding, the tools were ground to their original shape and their weight was measured. Subsequently, the tool was sawn, and specimens were created for Rockwell hardness evaluation, material microstructure and for abrasive wear resistance testing as per ASTM G133-95. The OK WearTrode 55 electrode is a hardfacing electrode that produces weld metal with a high-volume fraction of fine carbides in a martensitic matrix. Better results were achieved by the UTP 690 hardfacing material. The hardness was 3.1 times higher compared to the base tool material 16MnCr5 and 1.2 times higher than the OK WearTrode 55 material. The abrasive wear resistance was 2.76 times higher compared to 16MnCr5, and 1.14 times higher compared to the OK WearTrode 55 material. The choice of a suitable pre-treatment for the tool and the selection and application of such additional material, which with its complex properties better resists the effects of the working environment, is a prerequisite for increasing the service life of tools working in forestry.

## 1. Introduction

As part of the global trend towards environmental protection in forestry and sustainable development of the forest environment, it has become an important issue to address the efficient use of natural resources through the use of modern, highly efficient and safe equipment in forest maintenance [1].

Crushing of unwanted growths is a certain method of mulching the woody mass of the growth of different types and thicknesses [2]. Mulching is widely recognized as an effective soil and water conservation measure all over the world. It is a mechanized type of work that destroys and crushes above-ground parts of vegetation. Globally, numerous laboratory and field studies have demonstrated that mulching is beneficial in reducing soil and water loss across a variety of environmental conditions, including agricultural lands [2,3,4]. Working bodies of agricultural machinery and equipment operate in harsh conditions. Mulching cutters shred various types of vegetation, including stumps, trees, bushes and their roots, and mix wood residues with soil, which acts as an abrasive [4]. Mulching devices are used as adapters that are clamped to the forest wheeled tractor. The main part of the adapter structure is a rotating cylinder with a rotation speed of n = 1000 min^−1^, which is attached to the base machine. Tools are placed around the circumference of the rotary cylinder [5,6]. These machines can work even in broken terrains, with large slopes. They are often used in forestry to process unwanted growth, especially in inaccessible terrain under high voltage lines, etc.

The wear and tear of mechanisms and machine parts is a phenomenon constantly accompanying the operation of any machinery, and it causes measurable financial losses [7,8]. Deterioration processes usually begin in the superficial layer of a given part. The consequence of wear is a progressive loss of mass and initial, nominal dimensions [9,10]. The fastest wearing part of the assembly is the tools. Tools for crushing unwanted growth operate in a strongly heterogeneous environment. Due to the impact of the working environment, in which there are various types of wood, soil, minerals and rocks, they are subject to high wear. This wear is caused by the impact and abrasion of hard abrasive particles. Abrasive wear is believed to be responsible for up to 50% of failures, or the replacement of parts, components and tools [11]. After the loss of the wolfram–carbide WC tip, there is a rapid loss of material precisely on these surfaces, which leads to a total loss of its function, namely the crushing of unwanted growth. These tools are also subject to impact load. The results of long-term research by the authors [5,12,13,14] show that the observed wear of the material is caused by cyclic plastic deformation. Strengthening of the material before increased stress, which is a manifestation of cold plastic deformation, increased its hardness, and therefore also its brittleness [15]. Cyclic repetition of the plastic deformation of the tool under abrasion conditions leads to a loss of material, mainly on the back surface of the tool, over time (after the loss of WC tips).

The working bodies of the mulching cutter are made of different materials depending on the requirements for strength, wear resistance, corrosion resistance and other properties. One of the most common materials is high-quality carbon steel. Steels with the addition of chromium or tungsten can also be used to improve performance. Another popular material for the production of mulching cutter workpieces is carbide materials such as tungsten carbide, which have high hardness and wear resistance. Materials based on ceramics or composites can also be used [4,12,16].

There are several methods for increasing the service life of tools—heat, chemical heat treatment, hardfacing by welding, etc [17]. Based on long-term research in the field of increasing the service life of tools for crushing unwanted growths, the tools were modified through heat treatment. However, the results showed that the hardness of the modified tool (with 16MnCr5 material) was 1.75 times higher than the hardness of the unmodified tool, but at the same time, the performed heat treatment had almost no effect on the resistance to abrasive wear [5]. One of the very effective measures for increasing wear resistance is the application of a suitable additional material on functional surfaces [18]. Despite being used for over 100 years, there still is much to discover, as modern metallurgy provides more and more sophisticated alloys, which then have to be studied to find the best technological parameters in order to fully utilize complex material properties [7,18,19,20]. Hardfacing is a commonly used method to improve the surface properties of agricultural tools, components for mining operations, soil treatment equipment and others [21,22,23]. However, it is still not widely used for wood processing applications in forestry. The alloy is homogeneously applied to the surface of the base material (usually low-carbon or medium-carbon steels) by hardfacing in order to increase hardness and wear resistance without significant loss of ductility and toughness of the base material [13,23,24,25]. According to the author Kiswel [3], K-700HT flux-cored wire or welding flux-cored wire VELTEK-N565, which is used for hardfacing of mining equipment, impact drilling bits, screens, augers and parts of high-manganese austenitic steels, providing a hardness of 54 to 60 HRC [4].

The main components of Fe-based alloys are chromium, molybdenum and boron, which make Fe-based alloys resistant to wear. Wang et al. [25] studied the microstructure, hardness and wear resistance of Fe-based alloys by adding the elements ferrotitanium (Fe-Ti), ferromolybdenum (Fe-Mo), ferrovanadium (Fe-V) and graphite, which were applied by the arc welding process [26]. The results showed that the hardness and wear resistance of the hardfacing metal layer increased with an increasing proportion of Fe-Ti, Fe-V, Fe-Mo and graphite. Their amounts must be controlled in the range of 8–10% graphite, 12–15% Fe-Ti, 10–12% Fe-V and 2–4% Fe-Mo. However, cracks begin to form in the layer of hardfacing metal if the amount of Fe-Mo is more than 5% [27,28]. Important factors that determine the resistance to abrasive wear include the hardness, size, shape and intensity of the particles, and the hardness, shape, size and number of hard phases and their distribution in the base metal. Some authors [25,28,29] state that wear resistance increases with the increasing hardness of hard structural elements (carbides, borides, etc.) and their increasing share in the structure. During impact wear, the material is exposed to impact and high pressure, due to which it deforms or locally breaks—it can even crack.

Previous research has not considered that by hardfacing through welding the additive material to the surface of the tool, the weight of the tool will be increased. As several of these tools are mounted on the rotor of the base machine adapter (depending on the rotor dimensions, approx. 30 to 50 tools), this change in tool weight will also change the rotor loading dynamics, which may also affect the mulching process itself. In order to avoid an increase in tool weight, it is necessary to remove material from the tool, which will then be replaced in the next step by being welded on an additional material. At the same time, the shape of the tool must also be preserved, so it is necessary to grind the tool to its original shape after hardfacing by welding. To improve the surface, new machining methods have been developed in recent years. As the authors of [30] state in their research, a new body-armor-like abrasive tool (BAAT) with a soft and hard combined substrate (metal–elastomer substrate) to achieve high-shear and low-pressure grinding, it is possible to almost completely remove surface residual defects such as pits and deep scratches under optimal conditions. Also, the surface quality was significantly improved after grinding [30]. In their research, to prepare a flexible grinding body-armor-like abrasive tool (BAAT) with an increased tangential force-to-normal-force ratio, Wei. et al. found that smaller grinding grain sizes generated lower surface roughness values in zirconia ceramics after grinding [31].

The aim of this research is to increase the resistance to abrasive wear as well as the hardness of the tool body, because after a very short time, these tools need to be changed, which causes frequent machine downtime. Also, the efficiency and quality of the mulching process is reduced when using tools are worn out. At the same time, these tools are quite expensive—the price ranges up to EUR 100. This causes both technical and economic problems for companies operating in forestry. For this reason, it is necessary to look for solutions to increase the service life of these tools.

## 2. Materials and Methods

**Figure 1:**
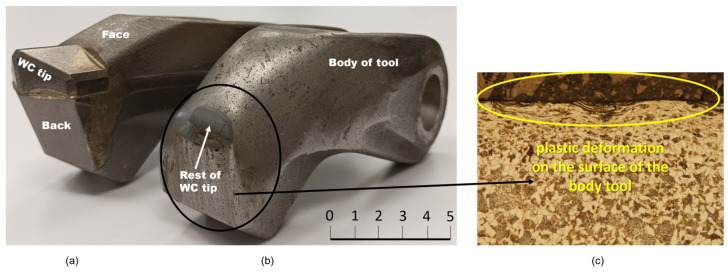
New, unused tool (**a**); damaged, non-functional tool (**b**) and microstructure of material with plastic surface deformation (**c**) [2,14].

The two areas on the tool that are most stressed during its operation were identified, namely the face and back of the tool (Figure 1a). Since the tool operates in a highly heterogeneous environment, after a short time, the WC tip is lost and there is a significant and rapid loss of material on the face and back of the tool (Figure 1b). Figure 1c shows a plastically deformed surface with traces of pressed-in abrasive elements from the working environment of the tool. The ferritic–pearlitic structure cannot withstand such a significant abrasive load as the tool body is subjected to.

The body of the tools for crushing unwanted growths is made of 16MnCr5 (according to STN EN ISO 683-3:2022) [5,32], which is a stainless structural manganese–chromium steel designed for cementing. The steel is well ductile in hot annealing and after soft and cold annealing, and is easy to machine and weld. It is ideal for machine components that are subsequently hardened to a diameter of 35 mm and is suitable for high core strength cementing. It is used for the manufacturing of components such as shafts, gears, camshafts, valve lifters, piston pins and gear couplings.

The most exposed areas of the tool need to be strengthened, which is supposed to increase the resistance of the tool to the loss of the WC tip and also to increase the life of the tool even after the loss of the WC tip. The novelty lies in the modification of the (new) tool by removing material from the face and back. This removed material is then replaced by a new material—an additional hardfacing material which, due to its chemical composition and structure, is better able to withstand the effects of the working environment. At the same time, this pre-preparation ensures that the weight and shape of the tool are maintained.

To verify the suitability of the choice of hardfacing materials, it is necessary to carry out laboratory tests, which include the following:

- Rockwell hardness measurement,
- Microstructure assessment by SEM microscopy,
- Abrasive wear resistance test—in principle ball-on-flat.

### 2.1. Tool Modification Before Hardfacing by Welding

**Figure 2:**
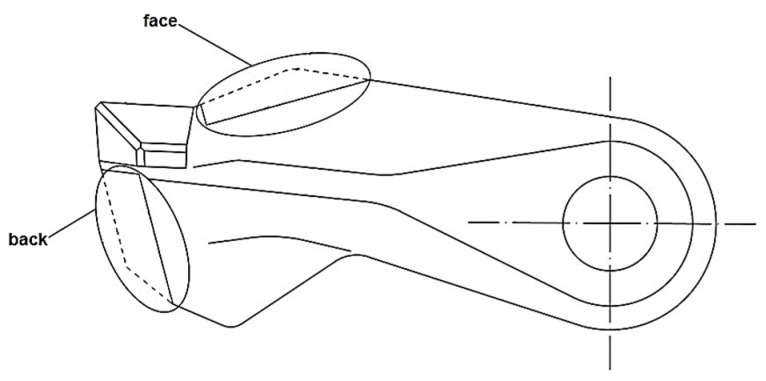
Modified tool surfaces.

When the additive material is hardfaced onto the exposed surfaces of the functional parts of the tools without prior preparation, the overall weight of the tool increases and the shape of the tool changes. This process will also affect the loading dynamics of the rotor to which the tools are mounted. Such a change can become one of the causes of faster tool wear. One way to prevent this is to pre-prepare the exposed surfaces of the tools by removing material from the tool body. Modifications were carried out on the face and back of the tool (Figure 2).

**Figure 3:**
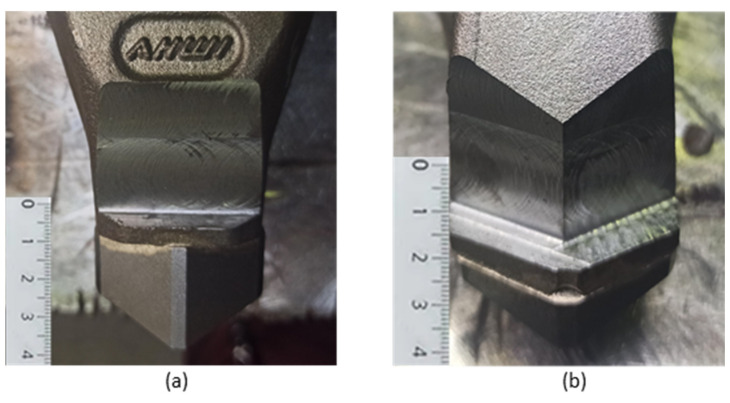
Modified face of tool (**a**); modified back of tool (**b**).

The tool surfaces were prepared by chip machining—milling with a face mill with a diameter of 18 mm. A 20 × 30 mm surface was milled on the face of the tool (Figure 3a) and, 20 × 15 mm two surfaces were milled on the back (Figure 3b) to maintain the shape of the tool. The milling was carried out to a depth of 5 mm.

### 2.2. Hardfacing Materials and the Hardfacing Process

The selected hardfacing materials were subsequently hardfaced onto the pre-prepared surfaces. These hardfacing materials should provide better wear resistance due to their chemical composition and structure. The hardfacing material was applied to each tool in two layers. After the first layer of hardfacing material was applied, the hardfaced surfaces were cleaned and the second layer of hardfacing material was applied in a direction rotated by 90° with respect to the direction of the first layer of hardfacing material.

The manual arc hardfacing (MMA) method was used for the experiment. Two hardfacing electrode materials were chosen, namely UTP 690 (STN EN 14700: E Fe4 [33], Bohler) and OK WearTrode 55 (STN EN 14700: E Z Fe6 [33], Esab). The chemical composition of these electrodes is in Table 1. The characteristics of hardfacing electrodes are as follows:

- The UTP 690 is used for the repair and production of cutting tools, especially for the restoration of cutting edges and working surfaces. The hardfacing metal is highly resistant to friction, compression and impacts, even at elevated temperatures of up to 550 °C. Using this electrode, it is also possible to manufacture new tools by hardfacing on unalloyed and low-alloy base steels. After hardfacing, it forms a martensitic structure. The weld deposit is equivalent to a high-speed steel with increased Mo content. The type of electrode coating is rutile. The hardness of the coating in the second layer is 62 HRC [34].
- The OK WearTrode 55 is a high-stress electrode for welding wear-resistant functional surfaces under simultaneous impact stresses with the necessary partial corrosion resistance. Machining of the coating is possible by grinding. After welding, it forms a martensitic structure [19,35]. It is used as a coating for parts of agricultural and forestry machinery, mixers, transport equipment, etc. The type of electrode coating is lime-basic. The hardness of the coating in the second layer is 52–59 HRC [35].

The tools were hardfaced by certified welders in the company ŽOS, a.s. in Zvolen, Slovakia. Electrodes with a diameter of 2.5 mm was used for hardfacing by welding. The hardfacing surfaces were cleaned manually with a wire brush and degraded with an industrial solvent. When hardfacing by welding with UTP 690 electrodes, the electrodes were dried at 300 °C for 2 h in a Zepacomp drying oven. The tool was preheated to a temperature of 250 °C. The welding current was set to I = 90 A and the voltage U = 24.5 V. During the hardfacing by welding with OK WearTrode 55 electrodes, the electrodes were dried at 200 °C for 2 h in a Zepacomp drying oven. The tool was preheated to 200 °C. The set welding current was I = 90 A and voltage U = 24.5 V.

**Figure 4:**
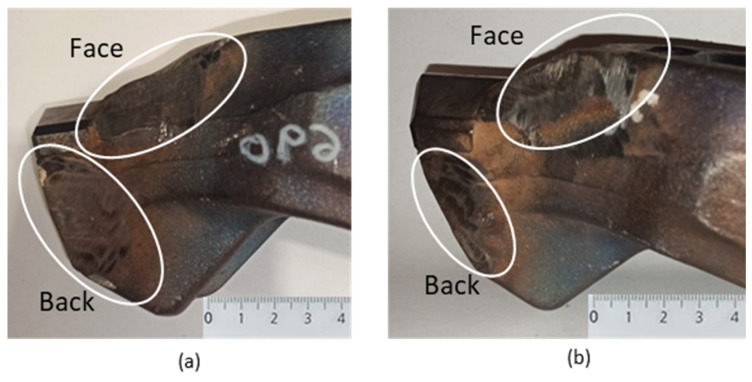
Tool after grounding of hardfacing: UTP 690 hardfacing material (**a**); OK WearTrode 55 hardfacing material (**b**).

It was important to minimize the heat input during the hardfacing by welding process. After each 20 mm section, it was necessary to measure the interpass temperature using a touch thermometer. The interpass temperature was prescribed by the manufacturer at 200 °C. If the specimen exceeded this temperature, it was necessary to wait until the specimen temperature dropped below the specified interpass temperature to proceed with the surfacing. The tool was always cleaned during the hardfacing process to prevent inclusions from impurities in the weld metal. After steaming, the samples were allowed to cool freely in air to room temperature and then cleaned. Figure 4a shows the tool with UTP 690 and Figure 4b shows the tool with OK WearTrode 55.

After the additional materials were hardfaced onto the pre-prepared tool, the hardfacing material was ground to maintain the shape of the tool. Figure 4a,b show the tool after modification—the material was removed from the face and back of the tool with the subsequently hardfaced and grounded additive material UTP 690 (Figure 4a) and OK WearTrode 55 (Figure 4b).

### 2.3. Evaluation Methods

In order to evaluate the suitability of the selected hardfacing electrodes, laboratory analyzes were performed on the samples:

- Measurement of the weight of the tools before and after modification on the AG2000C digital scale (Axis 4 Sp. z o.o., Gdańsk, Poland).
- Rockwell hardness measurement according to ISO 6508-1 on universal hardness tester with max. load 250 kg, model UH250 (Bauhler, Boston, MA, USA) [37].
- Evaluation of the microstructure of the tool body material and additional hardfacing materials on an Olympus GX71 metallographic microscope with an Olympus DP12 camera (Olympus, Tokyo, Japan). Samples for microstructure evaluation were prepared in a standard method. To develop the structure, 2% Nital etchant (2% HNO_3_ solution in ethyl alcohol) was used for the base material and Cor etchant (120 mL CH_3_COOH, 20 mL HCl, 3 g picric acid, 144 mL CH_3_OH) was used for the hardfacing materials.
- Test of resistance to wear, performed according to ASTM G133-95 with macroscopic evaluation of traces after the test on a Zeiss Stemi 2000 stereomicroscope (Carl Zeiss AG, Oberkochen, Germany) [38].
- Wear of a flat sample with determination of the volume V_f_, evaluated with a 3D profilometer TalySurf CLI 1000 with a confocal and touch induction sensor (Taylor Hobson, Leicester, UK).

The wear resistance test was performed according to the ASTM G133-95 Standard Test Method for Linearly Reciprocating Ball-on-Flat Sliding Wear. This test method covers laboratory procedures for determining the sliding wear of ceramics, metals, and other candidate wear-resistant materials using a linear, reciprocating ball-on-flat plane geometry. The direction of the relative motion between sliding surfaces reverses in a periodic fashion such that the sliding occurs back and forth and in a straight line. The principal quantities of interest are the wear volumes of the contacting ball and flat specimen materials. The load is vertically downward through the spherical specimen against a horizontally mounted flat-shaped specimen. Dimensional changes for both ball and flat specimens are used to calculate wear volume and wear rate. The tangential force can be measured continuously during the oscillating contact and serves to obtain data on the coefficient of friction [38].

**Figure 5:**
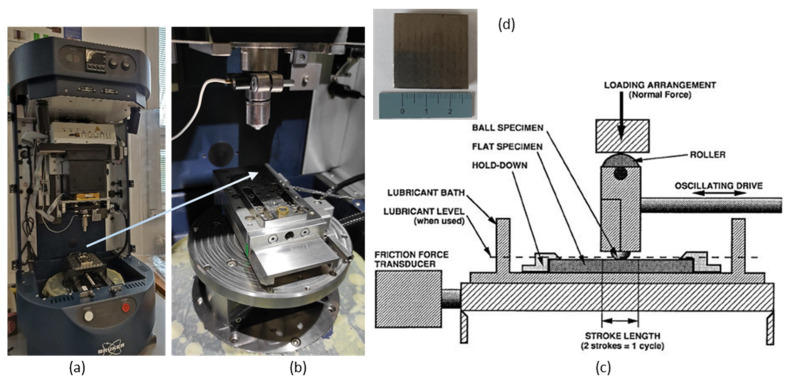
Bruker TriboLab instrument (**a**); detail of the workspace (**b**); schematic representation of the wear test apparatus [38] (**c**); tested sample (**d**).

In Figure 5a, the Bruker TriboLab instrument (Billerica, MA, USA) on which the test of resistance to abrasive wear was performed, with a detail of the working area of the instrument (Figure 5b) is shown. In Figure 5c is a schematic representation of the device [38]. In Figure 5d, the sample on which the test was performed is shown.

Flat sample wear—flat sample wear volume V_f_ is calculated from the stroke length and the average cross-sectional area of the wear track. The wear volume of a flat sample V_f_ (mm^3^) is calculated from Equation (1) [38]:

V_f_ = A · L (1)

where

A—average track cross-sectional area [mm^2^],
L—stroke length [mm].

The samples for the tests were prepared by abrasive water jet cutting technology. The dimensions of the samples were 30 mm × 30 mm × 5 mm (Figure 5d). The prepared sample was inserted into the device, and the time, load force and path of the ball movement along the flat sample were set. After the set time, a trace of the ball was observed on the sample.

The test conditions were set as follows:

- Loading force: 120 N,
- Frequency: 5 Hz,
- Test duration: 15,000 s,
- Track length: 10 mm,
- Ball material: sintered carbide (94% WC + 6% Co),
- Ball diameter: 6.35 mm,
- Hardness of the ball: 56 HRC.

## 3. Results and Discussion

Next, the results of the performed laboratory analyzes and tests on samples of the base material and hardfacing electrodes are presented for their evaluation of selected properties and determination of suitability for use in order to increase the service life.

### 3.1. Weight Measurement

In order to determine whether the weight of the tool changed after the modification, measurements of their weight before and after the modification were taken. Table 2 shows the measured weights of the instruments.

It can be concluded that the correct pre-preparation of the tool, followed by the hardfacing by welding of the additional material, does not lead to a significant change in weight.

### 3.2. Hardness Measurement

The measured hardness values according to Rockwell according to ISO 6508-1 [37] are in Table 3, five hardness measurements were taken on each sample and the average HRC value was calculated from them. 

Based on the results from the hardness measurements, we can conclude that both hardfacing materials achieved significantly higher hardness compared to the base tool body material 16MnCr5. Some authors report that hardness correlates strongly with abrasive wear resistance [25,39]. Thus, based on the results from hardness measurements, we can predict that the welds with the highest hardness will best resist the effects of an abrasive environment. However, other authors, in turn, have reported in their studies that hardness has no effect on wear resistance [13,40]. Therefore, it was necessary to carry out microstructure assessment and wear testing for the base material of the tool body and for the individual hardfacing materials.

### 3.3. Microstructure Evaluation

**Figure 6:**
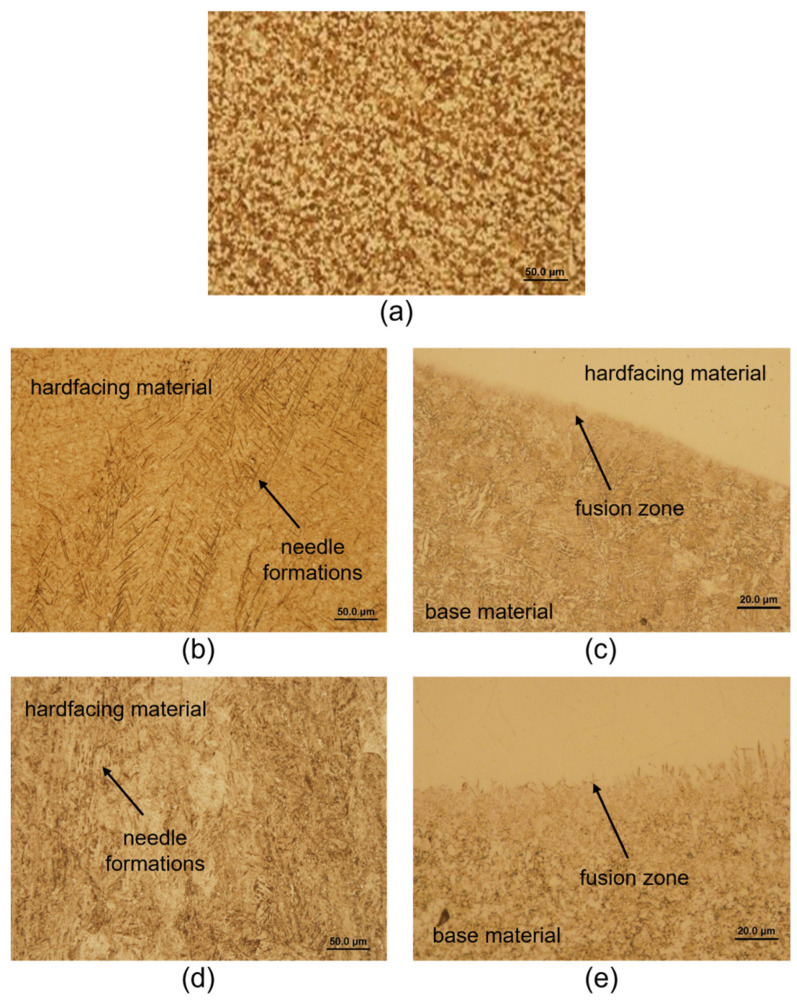
Microstructure of 16MnCr5 (**a**); microstructure of UTP 690 (**b**); fusion zone UTP 690-BM without observation of defects (**c**); microstructure of OK WearTrode 55 (**d**); fusion zone OK WearTrode 55-BM without observation of defects (**e**).

Figure 6a shows the microstructure of the tool body material 16MnCr5. It is a ferritic–pearlitic steel in a not-heat-treated state. Figure 6b shows the martensitic structure of the hardfacing material UTP 690. Needle-like formations are observed in the hardfacing metal of UTP 690, which indicates that this weld metal could better transfer the loads acting on it. In Figure 6c is a hard deposit layer of hardfacing material UTP 690 on the base material (BM). Their connection is without errors, no defects are observed in the remelting zone, which would predict an insufficient connection of the materials. There are no defects in the hardfacing metal (cracks, voids, inclusions, etc.). Figure 6d shows also martensitic structure of the OK WearTrode 55 hardfacing material. Figure 6e shows a hard deposit layer of the OK WearTrode 55 hardfacing material on the base material. There are also no defects, cracks or pores that would reduce the quality of the mixing of the base material with the hardfacing material.

### 3.4. Wear Resistance Test with Flat Specimen Wear Evaluation

**Figure 7:**
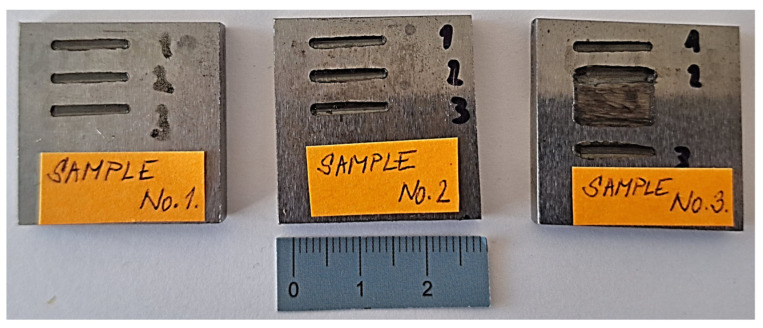
Samples after the wear resistance test (no. 1—base material, no. 2.—UTP690, no. 3—OK WearTrode 55).

Figure 7 shows the samples after the wear resistance test. Three tracks of straight lines were made on each sample.

**Figure 8:**
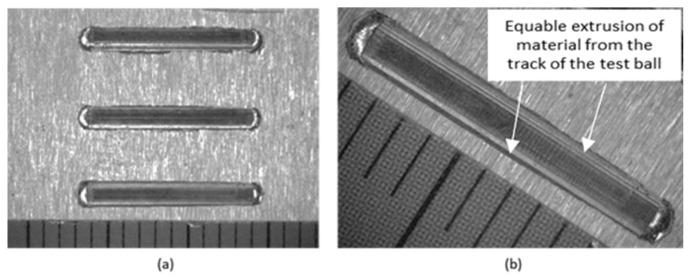
Tracks on sample no. 1—16MnCr5, mag.12.5× (**a**); track detail mag. 25× (**b**).

Macroscopic observation of the traces after the test was performed on a stereomicroscope. In Figure 8a, three tracks can be seen after the passage of the ball on sample no. 1 (tool body material 16MnCr5). From the details in Figure 8b, it can be seen that the track is uniform, the printed volume of material is uniform over the entire length of the track, a straight line.

**Figure 9:**
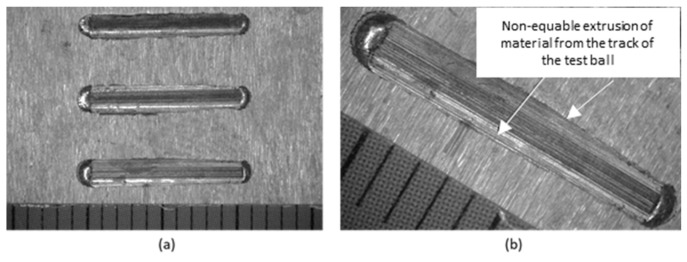
Tracks on sample no. 2—UTP 690, mag.12.5× (**a**); track detail mag. 25× (**b**).

In Figure 9a, there are three tracks of the ball on sample no. 2 (UTP 690). From the details in Figure 9b, it can be seen that the trace is not uniform. The pushed-out volume of material is larger on one side than on the other.

**Figure 10:**
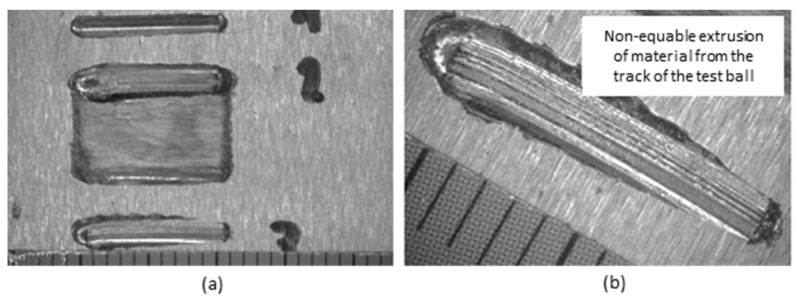
Tracks on sample no. 3—OK WearTrode 55, mag.12.5× (**a**); track detail mag. 25× (**b**).

Three tracks of the ball on sample no. 3 (OK WearTrode 55) are shown in Figure 10a. From the detail in Figure 10b, it can be seen that the track is not uniform. The pushed-out volume of material is larger on one side than on the other side of the track. However, we can conclude that the pushed-out material is more uniform than in sample no. 2.

**Figure 11:**
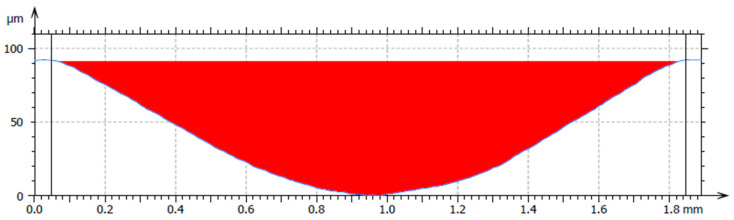
Average cross-sectional area of the 16MnCr5 track.

**Figure 12:**
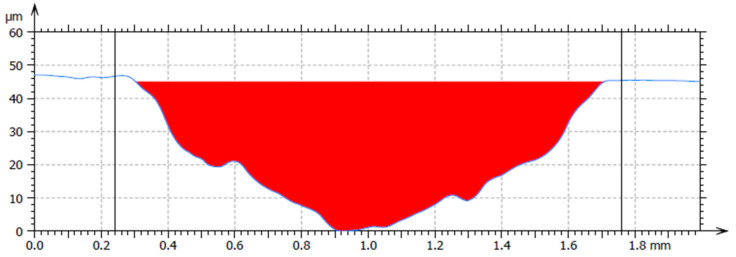
Average cross-sectional area of the UTP 690 track.

**Figure 13:**
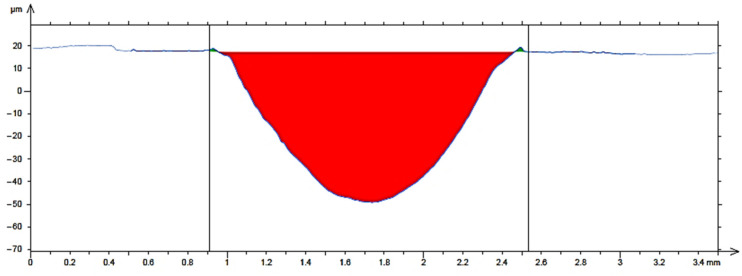
Average cross-sectional area of the OK WearTrode 55 track.

Figure 11 shows the average cross-sectional area of the trace for material 16MnCr5. For the hardfacing material UTP 690, the average cross-sectional area is shown in Figure 12, and for the hardfacing material OK WearTrode 55, that is shown in Figure 13. Here, it is possible to observe a more pronounced pushing-out of the material on both sides of the track (green areas).

Based on the measured data from individual measurements, the wear volumes of the flat sample were calculated for each material according to Equation (1) (Table 4).

**Figure 14:**
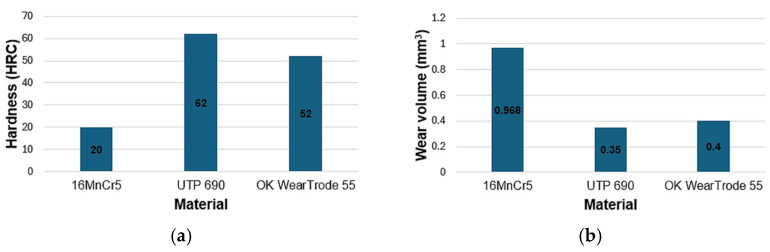
Graphs for comparing the values obtained in the experiment—hardness according to Rockwell (**a**); volume loss of material V_f_ (**b**).

Figure 14a shows a graph comparing the hardness of individual materials. The base material of the tool for crushing unwanted growths, 16MnCr5, reached an average hardness of 20 HRC. The hardfacing UTP 690 material reached an average hardness of 62HRC, and the OK WearTrode 55 reached an average hardness of 52 HRC. Compared to the base material, the hardfaced materials achieved significantly higher hardness, by more than three times. Some authors report that hardness is strongly correlated with resistance to abrasive wear [11,20,21,25,26,28]. However, the authors of [11,13,25] used different test methods than those used in this experiment to evaluate the resistance to abrasive wear. Figure 14b shows a graph comparing the values of the wear volume of the flat sample V_f_ for each material tested. The lowest volume loss was achieved by UTP 690, namely 0.350 mm^3^. Compared to OK WearTrode 55, this is only 0.05 mm^3^ more, even though the hardness of UTP 690 was 10 HRC higher. The base material 16MnCr5 reached the highest volume loss value, namely 0.968 mm^3^.

The hardfacing material UTP 690, with its chemical composition represented by chemical elements Cr and W, also increased in hardness and wear resistance. The elements V and W refined the grain. These elements contribute to increasing abrasion resistance. Vanadium increased the toughness needed to balance the increased hardness with the carbides C, Cr and W. The chemical composition of the OK WearTrode 55 hardfacing material is richer in Cr, but elements such as Mo, V and W are absent. Figure 6 shows images from a light microscope, showing the structures of individual materials. Both hardfacing materials have a martensitic structure with the presence of needle-like formations, which could make these hardfacing materials better able to transfer the loads acting on it; therefore, it is assumed that they will better withstand the effects of an abrasive environment. As stated by some authors [9,19,21], the microstructure of the material also has a great influence on wear resistance.

## 4. Conclusions

Based on the results of experiments carried out in laboratory conditions and presented in the article on two selected hardfacing materials UTP 690 and OK WearTrode 55 applied to the base material 16MnCr5, we can conclude that:

- Appropriate pre-treatment of the tool can ensure that its weight is maintained after the additional material has been hardfaced by welding.
- The HRC hardness of both hardfacing materials reached a higher average value than the hardness of the base material 16MnCr5.
- Both hardfacing materials achieved significantly better results compared to the base material of the 16MnCr5 tool—the UTP 690 coating achieved 2.76 times higher and the OK WearTrode 55 coating achieved 2.42 times higher abrasion wear resistance than the 16MnCr5 tool body material.
- Comparing the hardfacing materials with each other, better results were achieved by the UTP 690 hardfacing material: resistance to abrasive wear was 1.14 times higher compared to OK WearTrode; also, its hardness reached 1.19 times higher value.

In order to confirm the results of the laboratory measurements and tests, efforts will be made to make the given modifications to the tools that will be tested under operational conditions.

## Figures and Tables

**Table 1:** Standard chemical composition (mass.%) of the base material and electrodes [34,35,36].

	C	Si	Mn	Cr	Mo	P	S	V	W	Ti	Fe
16MnCr5	0.14–0.19	0.1–0.37	1.1–1.4	0.8–1.1	-	max. 0.035	max. 0.035	-	-	-	balance
UTP 690	0.9	0.8	0.5	4.5	8.0	-	-	1.2	2.0	-	balance
OK WearTrode 55	0.7	0.6	0.7	10.0	-	-	-	-	-	-	balance

**Table 2:** Weight of tools.

	New Unmodified Tool	Modified Tool
**UTP 690**	1730 g	1715 g
**OK WearTrode 55**	1730 g	1714 g

**Table 3:** Hardness values according to Rockwell.

		Values HRC					
		1.	2.	3.	4.	5.	Average
**Sample No. 1**	16MnCr5	20	21	20	19	20	20 ± 0.71
**Sample No. 2**	UTP 690	60	61	62	62	63	62 ± 1.14
**Sample No. 3**	OK WearTrode 55	50	52	52	51	53	52 ± 1.14

**Table 4:** Data obtained from the test of resistance to wear.

Sample		Max. Track Width (mm)	Max. Track Depth (mm)	Track Cross-Sectional Area Content (mm^2^)	Wear Volume of Flat Sample (mm^3^)
**Sample No. 1**	16MnCr5	1.790	0.0910	0.0968	0.968
**Sample No. 2**	UTP 690	1.450	0.0441	0.0350	0.350
**Sample No. 3**	OK WearTrode 55	1.507	0.0440	0.0350	0.400

## Data Availability

The original contributions presented in this study are included in the article. Further inquiries can be directed to the corresponding author.
